# COVID-19-associated acute kidney injury: consensus report of the 25th Acute Disease Quality Initiative (ADQI) Workgroup

**DOI:** 10.1038/s41581-020-00356-5

**Published:** 2020-10-15

**Authors:** Mitra K. Nadim, Lui G. Forni, Ravindra L. Mehta, Michael J. Connor, Kathleen D. Liu, Marlies Ostermann, Thomas Rimmelé, Alexander Zarbock, Samira Bell, Azra Bihorac, Vincenzo Cantaluppi, Eric Hoste, Faeq Husain-Syed, Michael J. Germain, Stuart L. Goldstein, Shruti Gupta, Michael Joannidis, Kianoush Kashani, Jay L. Koyner, Matthieu Legrand, Nuttha Lumlertgul, Sumit Mohan, Neesh Pannu, Zhiyong Peng, Xose L. Perez-Fernandez, Peter Pickkers, John Prowle, Thiago Reis, Nattachai Srisawat, Ashita Tolwani, Anitha Vijayan, Gianluca Villa, Li Yang, Claudio Ronco, John A. Kellum

**Affiliations:** 1 Division of Nephrology and Hypertension, Department of Medicine, Keck School of Medicine, University of Southern California, Los Angeles, CA USA; 2 Department of Clinical and Experimental Medicine, Faculty of Health Sciences, University of Surrey, Guildford, UK; 3 Intensive Care Unit, Royal Surrey County Hospital NHS Foundation Trust, Guildford, UK; 4 Division of Nephrology, Department of Medicine, University of California, San Diego, CA USA; 5 Divisions of Pulmonary, Allergy, Critical Care, & Sleep Medicine, Division of Renal Medicine, Department of Medicine, Emory University School of Medicine, Atlanta, GA USA; 6 Divisions of Nephrology and Critical Care Medicine, Departments of Medicine and Anesthesia, University of California, San Francisco, CA USA; 7 Department of Intensive Care, Guy’s & St Thomas’ NHS Foundation Hospital, London, UK; 8 Department of Anesthesiology and Intensive Care Medicine, Edouard Herriot Hospital, Hospices Civils de Lyon, Lyon, France; 9 Department of Anesthesiology, Intensive Care and Pain Medicine, University Hospital Münster, Münster, Germany; 10 Division of Population Health and Genomics, School of Medicine, University of Dundee, Dundee, UK; 11 Department of Medicine, University of Florida, Gainesville, FL USA; 12 Nephrology and Kidney Transplantation Unit, Department of Translational Medicine, University of Piemonte Orientale, Novara, Italy; 13 Intensive Care Unit, Ghent University Hospital, Ghent University, Ghent, Belgium; 14 Division of Nephrology, Pulmonology and Critical Care Medicine, Department of Medicine II, University Hospital Giessen and Marburg, Giessen, Germany; 15 Division of Nephrology, Renal Transplant Associates of New England, Baystate Medical Center U Mass Medical School, Springfield, MA USA; 16 Division of Nephrology and Hypertension, Cincinnati Children’s Hospital Medical Center, Cincinnati, OH USA; 17 Division of Renal Medicine, Brigham and Women’s Hospital, Boston, MA USA; 18 Division of Intensive Care and Emergency Medicine, Department of Medicine, Medical University of Innsbruck, Innsbruck, Austria; 19 Division of Nephrology and Hypertension, Division of Pulmonary and Critical Care Medicine, Department of Medicine, Mayo Clinic, Rochester, MN USA; 20 Division of Nephrology, Department of Medicine, University of Chicago, Chicago, IL USA; 21 Department of Anesthesiology and Perioperative Care, University of California San Francisco, San Francisco, CA USA; 22 Division of Nephrology, Excellence Center for Critical Care Nephrology, Critical Care Nephrology Research Unit, King Chulalongkorn Memorial Hospital, Chulalongkorn University, Bangkok, Thailand; 23 Department of Medicine, Division of Nephrology, Columbia University College of Physicians & Surgeons and New York Presbyterian Hospital, New York, NY USA; 24 Department of Epidemiology, Mailman School of Public Health, Columbia University, New York, NY USA; 25 Division of Critical Care Medicine, Faculty of Medicine and Dentistry, University of Alberta, Edmonton, Alberta Canada; 26 Division of Critical Care Medicine, Zhongnan Hospital of Wuhan University, Wuhan, China; 27 Servei de Medicina Intensiva, Hospital Universitari de Bellvitge, L’Hospitalet de Llobregat, Barcelona, Spain; 28 Department of Intensive Care Medicine, Radboudumc, Nijmegen, The Netherlands; 29 Critical Care and Peri-operative Medicine Research Group, William Harvey Research Institute, Queen Mary University of London, London, UK; 30 Department of Nephrology, Dialysis and Transplantation, San Bortolo Hospital, International Renal Research Institute of Vicenza, Vicenza, Italy; 31 Department of Nephrology, Clínica de Doenças Renais de Brasília, Brasília, Brazil; 32 Academy of Science, Royal Society of Thailand, Bangkok, Thailand; 33 Division of Nephrology, Department of Medicine, University of Alabama, Birmingham, AL USA; 34 Division of Nephrology, Washington University School of Medicine, St. Louis, MO USA; 35 Section of Anaesthesiology and Intensive Care, Department of Health Science, University of Florence, Florence, Italy; 36 Renal Division, Peking University First Hospital, Beijing, China; 37 Department of Medicine, University of Padova, Padova, Italy; 38 Department of Critical Care Medicine, Center for Critical Care Nephrology, University of Pittsburgh, Pittsburgh, PA USA

**Keywords:** Infectious diseases, Scientific community, Acute kidney injury, SARS-CoV-2

## Abstract

Kidney involvement in patients with coronavirus disease 2019 (COVID-19) is common, and can range from the presence of proteinuria and haematuria to acute kidney injury (AKI) requiring renal replacement therapy (RRT; also known as kidney replacement therapy). COVID-19-associated AKI (COVID-19 AKI) is associated with high mortality and serves as an independent risk factor for all-cause in-hospital death in patients with COVID-19. The pathophysiology and mechanisms of AKI in patients with COVID-19 have not been fully elucidated and seem to be multifactorial, in keeping with the pathophysiology of AKI in other patients who are critically ill. Little is known about the prevention and management of COVID-19 AKI. The emergence of regional ‘surges’ in COVID-19 cases can limit hospital resources, including dialysis availability and supplies; thus, careful daily assessment of available resources is needed. In this Consensus Statement, the Acute Disease Quality Initiative provides recommendations for the diagnosis, prevention and management of COVID-19 AKI based on current literature. We also make recommendations for areas of future research, which are aimed at improving understanding of the underlying processes and improving outcomes for patients with COVID-19 AKI.

## Abstract (web summary)

COVID-19-associated AKI (COVID-19 AKI) is associated with high mortality and is an independent risk factor for all-cause in-hospital death in patients with COVID-19. This Consensus Statement from the Acute Disease Quality Initiative provides recommendations for the diagnosis, prevention and management of COVID-19 AKI and for areas of future research, with the aim of improving understanding of the underlying processes and outcomes for patients with COVID-19 AKI.

## Introduction

A third novel coronavirus leading to severe respiratory infection (coronavirus disease 2019, COVID-19) was first identified in Wuhan, China in December 2019; as of August 2020, there have been 23 million confirmed cases with 800,000 deaths worldwide. The clinical spectrum resulting from infection with the responsible virus, severe acute respiratory syndrome coronavirus 2 (SARS-CoV-2), is broad, ranging from an asymptomatic response or development of a mild upper respiratory tract infection to critical illness. Initial reports of hospitalized patients in Wuhan described a high proportion of individuals with atypical pneumonia requiring critical care admission with features of acute respiratory distress syndrome (ARDS)^1,2^. The primary pulmonary pathology seemed to show not only diffuse alveolar damage but also evidence of direct viral cytopathy, implying a direct causative role of virus-induced damage in the development of ARDS rather than it resulting from a generalized inflammatory response.

Initial reports also indicated that rates of acute kidney injury (AKI) were negligible^2–7^. However, growing evidence has demonstrated that AKI is in fact prevalent among patients with COVID-19, particularly among patients in the intensive care unit (ICU)^8–19^. The reported rates of AKI are extremely variable; however, available evidence suggests that it likely affects >20% of hospitalized patients and >50% of patients in the ICU^8–19^. Similar to the association of AKI with other forms of community-acquired pneumonia^20^, AKI is now recognized as a common complication of COVID-19. As with AKI from other causes, COVID-19-associated AKI (COVID-19 AKI) is associated with adverse outcomes, including the development or worsening of comorbid disease as well as greater use of health-care resources. However, despite considerable advances in our understanding and management of other forms of AKI, relatively little is known about the pathogenesis or optimal management of COVID-19 AKI.

Given the paucity of knowledge with regard to optimal prevention and treatment strategies for COVID-19 AKI, the 25th Consensus Conference of the Acute Disease Quality Initiative (ADQI) focused on this new disease entity. The process was aimed at reviewing the current literature relating to COVID-19 AKI, including its epidemiology and pathophysiology, and compare our current understanding with that of other forms of AKI, with the aim of providing recommendations for the diagnosis, prevention and treatment of COVID-19 AKI. In addition to recommendations for the provision and delivery of renal replacement therapy (RRT; also known as kidney replacement therapy), we also explored the role of other extracorporeal techniques.

## Methods

The Conference Chairs of the 25th ADQI consensus committee (M.K.N, L.G.F, R.L.M., C.R. and J.A.K.) convened a diverse panel of clinicians and researchers representing nephrology and critical care from the Americas, Europe and Asia to discuss the issues relating to AKI associated with COVID-19. The conference was held virtually, over 4 weeks, with weekly virtual consensus meetings from 23 May to 13 June 2020. This consensus meeting followed the established ADQI process, and used a modified Delphi method to achieve consensus, as previously described^21^.

Conference participants were divided into five workgroups (Supplementary Box 1), which were tasked with addressing the following themes that are central to AKI in this patient population: pathophysiology and effects of treatments on the kidney; epidemiology and diagnosis; prevention and management; RRT, particularly under conditions of increased demand (surge); and the use of other forms of extracorporeal blood purification (EBP) for patients with COVID-19 with or without AKI. Members of the workgroups developed core questions, performed systematic literature reviews and developed a consensus, backed by evidence where possible, to distil the available literature and articulate a research agenda to address important unanswered questions. Literature searches were performed using the National Institutes of Health (NIH) COVID-19 portfolio and PubMed. Where possible, delegates were asked to note the level of evidence for consensus statements using the Oxford Centre for Evidence-based Medicine Levels of Evidence^22^. All of the individual workgroups presented their output to conference participants during the four videoconference plenary sessions for debate, discussion, suggested revisions, and the final product was then assessed and aggregated in a videoconference session attended by all attendees, who approved the consensus recommendations.

## Pathophysiology and effects of treatment

### Direct pathogenic mechanisms

*What direct pathogenic mechanisms have been implicated in COVID-19 AKI?*

1. *Histopathological data are limited, but a wide range of pathological findings have been described in patients with COVID-19, in keeping with the idea that multiple causes of AKI exist, including those commonly found in critically ill patients*.
2. *SARS-CoV-2 might display viral tropism and directly affect the kidney*.
3. *Endothelial dysfunction, coagulopathy and complement activation are likely important mechanisms for AKI in a subset of patients with COVID-19*.
4. *The role of systemic inflammation and immune dysfunction in the development of COVID-19 AKI is still uncertain*.

#### Rationale

**Fig. 1:**
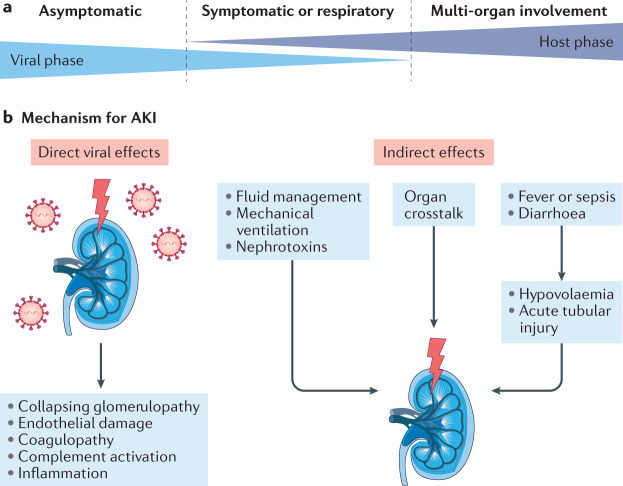
Pathogenesis of COVID-19 AKI. **a**,**b** | The pathogenesis of AKI in patients with COVID-19 (COVID-19 AKI) is likely multifactorial, involving both the direct effects of the SARS-CoV-2 virus on the kidney and the indirect mechanisms resulting from systemic consequences of viral infection or effects of the virus on distant organs including the lung, in addition to mechanisms relating to the management of COVID-19. AKI, acute kidney injury. Adapted from Acute Disease Quality Initiative 25, www.ADQI.org, CC BY 2.0 (https://creativecommons.org/licenses/by/2.0/).

Histopathological data relating to COVID-19 AKI are limited, but available evidence suggests that numerous causes of AKI exist in the setting of COVID-19 (Fig. 1). A post-mortem study of 26 patients who had died with COVID-19 AKI revealed prominent acute tubular injury on light microscopy^23^. In addition, this and another post-mortem study reported the presence of viral particles within both the tubular epithelium and podocytes on electron microscopy, implying direct infection of the kidney^23,24^. Collapsing glomerulopathy has also been described in patients with COVID-19. This morphological variant of focal segmental glomerulosclerosis is associated with a range of factors, including viral infection, particularly among patients of African ancestry, in whom the presence of high-risk *APOL1* alleles is a genetic risk factor for collapsing glomerulopathy, irrespective of cause^25–28^. However, not all reports are in agreement with regard to the pathological changes associated with COVID-19. For example, a biopsy study of a critically ill patient with COVID-19 demonstrated extensive acute tubular injury, yet real-time PCR on frozen kidney tissue, urine and serum were negative for SARS-CoV-2, and no evidence of direct viral kidney invasion was found^29^. Similarly, a post mortem study from Germany failed to demonstrate morphologically detectable changes in the kidney of patients who had died with COVID-19, although how many, if any, patients had AKI is unclear^30^. Further autopsy studies have also failed to demonstrate the presence of virus in the kidney^31^. These findings are in contrast to another study that documented SARS-CoV-2 RNA and protein in precisely defined, microdissected kidney compartments from autopsy specimens. Detectable SARS-CoV-2 viral load was demonstrated in all kidney compartments examined, with preferential targeting of glomerular cells^32^. In a second study from the same group, post-mortem data from patients who had COVID-19 respiratory infection demonstrated association of SARS-CoV-2 renal tropism with disease severity (that is, risk of premature death) and development of AKI^33^. Finally, whether pathological data will emerge to support a major role for thrombosis and microangiopathy in the kidney in patients with COVID-19 (as has been documented in the lung) remains to be seen^34,35^.

It is unknown but likely that certain genetic traits might increase susceptibility to COVID-19 AKI. The receptor-binding domain of the SARS-CoV-2 spike protein gains entry to host cells by binding to membrane-bound ACE2 — a protein that is also present on kidney tubular epithelial cells and podocytes^36,37^. Of note, limited data suggest that polymorphisms in *ACE2* might alter the ability of the virus to enter cells. As mentioned above, *APOL1* genotype might also affect disease susceptibility. Although the *APOL1* risk alleles do not themselves confer a high risk of kidney injury, a ‘second-hit’, such as that induced by SARS-CoV-2 infection, may lead to kidney injury^38,39^. Future investigations are needed to clarify these associations.

Endothelial dysfunction — characterized by high D-dimer levels and microvascular damage — represents a key risk factor for COVID-19-associated coagulopathy. Other inherited or acquired pro-thrombotic conditions, such as thrombotic thrombocytopenic purpura and atypical haemolytic uraemic syndrome might also contribute to endothelial dysfunction and coagulopathy in patients with COVID-19, as might direct viral activation of the complement system^40,41^. Importantly, complement activation and thrombotic microangiopathy are important mechanisms of kidney injury. However, so far, these have not been established as causing AKI in COVID-19.

Infection with SARS-CoV-2 is associated with activation of an inflammatory response that has been termed a ‘cytokine storm’, which might contribute to the pathogenesis of COVID-19-associated multi-organ dysfunction. However, what constitutes a cytokine storm remains ill-defined^42^. In other forms of viral respiratory illness caused by coronaviruses — severe acute respiratory syndrome (SARS; caused by SARS-CoV infection) and Middle East respiratory syndrome (MERS; caused by MERS-CoV infection) — high levels of pro-inflammatory cytokines were associated with more severe respiratory disease^43–45^. However, circulating levels of IL-6 reported for patients with COVID-19 are considerably lower than those observed in patients treated with chimeric antigen receptor T cell therapy; nearly 10,000-fold lower than those in patients with cytokine release syndrome, and >20,000-fold lower than those in patients with sepsis^46,47^. These comparisons indicate that cytokines are only moderately elevated in COVID-19 and are therefore unlikely to be directly pathogenic in most patients. An alternative explanation may be that these moderately elevated cytokines reflect the underlying critical illness, rather than representing a ‘storm’ of inflammatory processes per se. However, it is important to note that cytokine assays are not well standardized, and comparisons between studies using different assays are often not reliable. Cytokine levels, therefore, should be evaluated in larger cohorts of patients with COVID-19, as should concomitant analysis of immune cell count and activity. Understanding the role of innate and adaptive immune dysfunction in AKI patients should be a research priority.

### Indirect pathogenic mechanisms

*What are the indirect pathogenic mechanisms implicated in COVID-19 AKI?*

1. *Systemic effects of COVID-19 and critical care interventions may contribute to AKI*.
2. *Organ crosstalk is likely an important mechanism for AKI in patients with COVID-19*.
3. *Baseline patient characteristics contribute to AKI, acting as modifiers of direct pathogenic mechanisms*.

#### Rationale

In addition to direct pathophysiological mechanisms, renal dysfunction in the context of COVID-19 might also arise through the systemic effects of SARS-CoV-2 infection and critical illness (Fig. 1). For example, considerable insensible fluid losses might occur through hyperpyrexia and the gastrointestinal manifestations of COVID-19, such as diarrhoea, may result in volume depletion, an important potential contributor to AKI in other settings. Similarly, critically ill patients might be exposed to nephrotoxins as part of their clinical care, in particular, antibiotics, which can cause tubular injury or acute interstitial nephritis^48,49^. Moreover, individuals who develop secondary infections (regardless of whether they are bacterial, fungal or viral) are at increased risk of secondary sepsis-associated AKI^50^. Patients with severe COVID-19-associated pneumonia and/or ARDS are also at a high risk of AKI as a complication of mechanical ventilation. Specifically, COVID-19-associated ARDS is often treated by increasing positive end-expiratory pressure (PEEP), which leads to increased intrathoracic pressure and can ultimately result in increased renal venous pressure and reduced filtration, which may be further amplified if intra-abdominal pressure is elevated (for example, with fluid overload)^51^. In addition, all forms of positive pressure ventilation can increase sympathetic tone, leading to secondary activation of the renin–angiotensin system^52,53^. In the setting of ARDS after shock resolution, patients are often managed with a restrictive fluid strategy. However, in the setting of COVID-19, patients may initially present with relative volume depletion due to fever and gastrointestinal losses, and therefore careful attention to volume status is needed to avoid hypovolaemia.

Organ crosstalk describes the complex and mutual biological communication between distant organs mediated by signalling factors, including cytokines and growth factors, as well as the release of damage-associated molecular patterns (DAMPs) from injured tissue. Such crosstalk has also been suggested to mediate AKI in the setting of ARDS^54,55^.For example, lung injury in patients with COVID-19 can be severe and abrupt and lead to the release of not only DAMPs but also cytokines, chemokines and vasoactive substances that may continue to AKI. Tissues other than the lung might also serve as sources of DAMPs; for example, rhabdomyolysis in the setting of COVID-19 would result in the release of myoglobin from muscle^56^.

Available evidence suggests that older age, chronic kidney disease (CKD), and the presence of other comorbidities (for example, diabetes mellitus, hypertension, obesity, heart failure and chronic obstructive pulmonary disease) are associated with worse outcomes and also represent risk factors for the development of AKI in patients with COVID-19. These clinical features are characterized by low-grade inflammation and increased immune senescence, although how these impact the kidney in the setting of COVID-19 is unknown^57^.

### Recovery mechanisms

*What are the potential mechanisms for recovery from COVID-19 AKI?*

1. *Whether recovery from COVID-19 AKI differs from other forms of AKI is unknown. More research is needed to better understand the direct impact of the SARS CoV-2 virus on long-term renal fibrosis and recovery*.

#### Rationale

Pulmonary fibrosis in patients following recovery from COVID-19 has been reported^58^. Although we do not yet know whether kidney fibrosis occurs in patients who recover from COVID-19 AKI, the development of fibrosis and progression to CKD among patients who recover from other forms of AKI suggest that this scenario is likely. Moreover, the loss of functioning nephrons following injury might enhance the development of renal fibrosis. We recommend that patients with COVID-19 AKI be followed over a period of 2–3 months post-discharge, depending on the severity and acute needs of the patient, to evaluate kidney recovery^59^.

## Epidemiology and diagnosis

### Incidence and diagnosis

*What are the rates of COVID-19 AKI, and how should it be diagnosed?*

#### Recommendations

1. *Timing of AKI with symptom onset, hospitalization, confirmation of infection, disease severity and level of care should be characterized for appropriate clinical management (not graded)*.
2. *We recommend use of the Kidney Disease: Improving Global Outcomes (KDIGO) consensus definition for AKI, including serum creatinine (SCr) level and urine output, in clinical practice (evidence level: 1A)*.
3. *We suggest using kidney-specific tests along with measures of kidney function to characterize clinical presentations, course and outcomes of AKI (evidence level: 2B)*.

#### Rationale

Rates of reported AKI vary considerably between studies, with higher rates reported in countries outside of China (Table 1). Patients with COVID-19 may present with AKI or develop it during the course of their hospitalization. Those requiring admission to the ICU have a higher rate of AKI than those hospitalized on the wards. Of note, many published reports of patients with COVID-19 do not include definitions or staging of AKI, or information on renal recovery or follow up. The distinction between de novo AKI and AKI superimposed on pre-existing CKD is also rarely made. In addition, reported hospital admission rates of patients with COVID-19 varies between and within countries, reflecting different national and regional health-care systems, policies for hospitalization and for assigning levels of care (e.g. ICU admission). These factors all complicate comparisons of AKI rates based solely on the number of hospitalized patients. This variation is exemplified by data from the UK, where initial rates of AKI in April 2020 (as defined by need for RRT) were reported to be 20% in hospitalized patients with a reported mortality of over 80%, whereas data from July 2020 showed an incidence of 27% but an observed mortality of 57%^60^ (Table 1). Rather than reflecting an improvement in AKI outcomes, this variation reflects incomplete follow-up whereby more recent data from patients with shorter lengths of stay is not representative of the overall cohort. Such confounders should be considered when examining crude rates of AKI and the need for RRT.

**Table 1:** Rates of AKI and RRT in hospitalized patients with COVID-19

Study Population	*N*	ICU (%)	Comorbidities	AKI definition	AKI (%)	RRT (%)	Mortality in patients with AKI (%)	Ref
***China***								
Wuhan	116	0%	HTN: 37%; DM:16%; CKD: 4%	KDIGO	0%	4%	NR	^3^
Wuhan	99	23%	CVD: 40%; DM: 12%	SCr >1.3 mg/dl	3%	9%; 39% in ICU	NR	^4^
Wuhan	138	26%	HTN: 31%; DM: 10%; CKD: 3%	KDIGO	4%; 8% in ICU	2%; 6 % in ICU	NR	^2^
Wuhan	333	17%	HTN: 32%; DM: 23%	KDIGO	11% (46% stage1; 23% stage 2; 31% stage 3); 43% in ICU	3% in ICU	57%; 25% in stage 1; 75% stage 2; 90%; stage 3	^8^
Wuhan	701	10%	HTN: 33%: DM:14%; CKD: 2%	KDIGO	5% (2% stage 1; 1% stage 2; 2% stage 3)	NR	34% in patients with AKI on admission	^5^
Wuhan	41	32%	HTN: 15%; CVD: 15%; DM: 20%	KDIGO	7%; 23% in ICU	7%; 23% in ICU	NR	^65^
Wuhan	274	-	HTN: 34%; CVD: 8%; DM: 17%	KDIGO	11%	1%	NR	^9^
Wuhan	191	26%	HTN: 30%: DM: 19%; CKD: 1%	KDIGO	15%	5%	NR	^10^
Wuhan	52	100%	CVD: 23%; DM: 17%	KDIGO	29%	17%	NR	^11^
Wuhan	102	18%	HTN: 28%; CVD: 10%; DM: 11%; CKD: 4%	NR	20%	6%	NR	^12^
30 regions	1,099	5%	HTN; 15%; DM: 7%; CKD: 0.7%	KDIGO	0.5%; 6% in ICU	0.8%; 12% in ICU	NR	^6^
Jiangsu	80	0	CVD: 31%; CKD: 1%	NR	3%	1%	NR	^7^
***USA***								
Washington	21	100%	CKD: 48%; ESRD: 10%	Need for RRT	19%	NR	NR	^13^
New York	5700	22%	HTN 56%; CVD:18%; CKD: 5%; ESRD: 4%	KDIGO	24%	4%	NR	^14^
New York	1000	24%	HTN: 60%; CVD: 23%; DM: 37%; CKD: 14%	Defined by clinic notes in EHR	34%; 78% in ICU	14%; 35% in ICU	NR	^15^
New York	257	100%	HTN: 63%; CVD: 19%; DM: 36%; CKD: 14%	NR	NR	31%	NR	^68^
New York	5449	26%	HTN: 56%; CVD: 18%; DM: 33%	KDIGO	37% (47% Stage 1; 22 % Stage 2; 31% Stage 3); 76% in ICU	23% in ICU	35%	^16^
Louisiana	575	30%	HTN: 72%; DM: 48%; CKD: 29%	KDIGO	28%; 61% in ICU	15%; 73% in ICU	50%; 72% in patients on RRT	^17^
Multicentre	2215	100%	HTN: 60%; CVD: 22%; DM: 39%; CKD: 13%: ESRD: 3%	KDIGO Stage 2 and 3	43%	20%	NR	^19^
***Europe***								
United Kingdom	2,743 (April 2020) 10,547 (July 2020)	100%	CVD: 0.7%: ESRD: 2%	Need for RRT	NR	20% (April) 27% (July)	80% (April) 57% (July)	^18^

AKI, acute kidney injury; CKD, chronic kidney disease; CVD, cardiovascular disease; DM, diabetes mellitus; EHR, electronic health record; HTN, hypertension; ICNARC, Intensive Care National Audit & Research Center; ICU, intensive care unit; KDIGO, Kidney Disease Improving Global Outcomes; RRT, renal replacement therapy; SCr, serum creatinine; NR, not reported.

We recommend using KDIGO criteria for defining and reporting AKI^61^ to enable between-study comparison and because increased recognition of AKI through use of these criteria can improve survival^61,62^. Using these criteria, the epidemiology of COVID-19 AKI (Table 1) looks fairly similar to that of other forms of community-acquired pneumonia^20^. The lack of SCr measurements prior to hospital admission often impedes the ability to identify underlying CKD and creates challenges for the reliable detection and staging of AKI, emphasizing the need to define the baseline SCr clearly. One study in which baseline SCr measurements were available reported that 35% of patients with COVID-19 AKI had underlying CKD^17^. To improve understanding of the epidemiology and temporal nature of COVID-19 AKI, investigators must correlate the timing of AKI with COVID-19 symptom onset, hospitalization, confirmation of SARS-CoV-2 infection, disease severity and level of care when reporting AKI rates.

Although urine volume is reported infrequently, two-thirds of patients have low urinary sodium concentrations at the time of AKI, and the majority are oliguric at RRT initiation^16,17^. Urinalysis and biomarkers of AKI are frequently abnormal in patients with COVID-19 and could be used to characterize AKI in these patients^5,8,16,17^. For example, one study reported that among the 32% of patients hospitalized with COVID-19 for whom urinalysis was available, 42.1% had significant proteinuria, with leukocyturia and haematuria in 36.5% and 40.9%, respectively^16^. Similarly, a study of urinalysis data from 442 hospitalized Chinese patients with COVID-19, proteinuria was present in 43.9% (with 30% having ≥2+ on dipstick) with significant haematuria demonstrated in 11.3%^5^. Examination of urinary sediment can be an effective tool in clinical scenarios in which more than one possible cause of AKI may exist that could affect medical management, for example, to distinguish acute tubular necrosis from pre-renal AKI, although special precautions may be needed when handling biospecimens from patients with COVID-19 (ref.^63^). The role of urinary markers for injury or stress in the diagnosis of COVID-19 AKI remains unclear. Patients with COVID-19 AKI and high levels of tissue inhibitor of metalloproteinases-2 and insulin-like growth factor-binding protein-7 [TIMP-2] × [IGFBP-7] were more likely to progress to RRT than patients with AKI but with low [TIMP-2] × [IGFBP-7]^64^. Elevated urinary alpha1-microglobulin in hospitalized patients was associated with the subsequent development of AKI^64^. Patients with COVID-19 AKI have also been reported to have higher levels of systemic markers of inflammation, particularly ferritin, C-reactive protein, procalcitonin and lactate dehydrogenase, than patients with COVID-19 and normal kidney function^17^.

### Risk factors

*What are the risk factors for AKI with COVID-19 infection?*

#### Recommendations

1. *We suggest that patients be stratified for risk of AKI based on their comorbidities and demographics. Information about baseline CKD, comorbidities, and demographics should be obtained to define risk profiles of COVID-19 AKI* (Box 1) *(evidence level: 2C)*.
2. *Risk factors for community and hospital-acquired AKI, severity of illness, process of care, along with the threshold for admission to the hospital and the ICU should be considered when evaluating patients with COVID-19 (not graded)*.

Box 1 Potential Risk Factors for COVID-19 AKI **Demographic risk factors**

- Older age
- Diabetes mellitus
- Hypertension
- Cardiovascular disease or congestive heart failure
- High body mass index
- Chronic kidney disease
- Genetic risk factors (e.g. *APOL1* genotype; *ACE2* polymorphisms)
- Immunosuppressed state
- Smoking history

**Risk factors for AKI at admission**

- Severity of COVID-19
- Degree of viraemia
- Respiratory status
- Non-respiratory organ involvement, e.g. diarrhoea
- Leukocytosis
- Lymphopaenia
- Elevated markers of inflammation, e.g. ferritin, C-reactive protein, D-dimers
- Hypovolaemia/Dehydration
- Rhabdomyolysis
- Medication exposure, e.g. angiotensin-converting-enzyme (ACE) inhibitors and/or angiotensin-receptor blockers (ARBs), statins, nonsteroidal anti-inflammatory drugs (NSAIDs)

**Risk factors for AKI during hospitalization**

- Nephrotoxins (medications, contrast exposure)
- Vasopressors
- Ventilation, high positive end-expiratory pressure
- Fluid dynamics (fluid overload or hypovolaemia)

#### Rationale

Risk stratification is important to tailor monitoring and initiate prevention and/or early treatment strategies for patients who will benefit the most from intervention. Data from China and the USA suggest that male sex, older age, Black race, diabetes mellitus, CKD, hypertension, cardiovascular disease, congestive heart failure and higher body mass index are associated with COVID-19 AKI^8,16,17^. Among patients with COVID-19, those with AKI are more likely to require vasopressors as well as mechanical ventilation^16,17^. No data exist on criteria for ICU admission, or the association between medications and procedures (e.g. surgeries, contrast medium administration) and COVID-19 AKI.

AKI rates vary considerably between geographic regions and between different health systems. Data from China suggest that AKI is less common among patients in China^2–6,8–12,65,66^ than among patients in the USA^13,14,16,17,19,67,68^ and Europe^60^. This difference may be attributed to differences in the patient population studies; for example, patients in the Chinese studies had fewer comorbidities and were admitted to hospital with less severe respiratory disease or ARDS than patients in other cohorts (Table 1). To date, there are no data on risk factors for COVID-19 AKI between different hospital settings (for example, academic versus community hospitals, or rural versus urban hospitals) and although rapid surges in hospital admissions related to COVID-19 have been reported in New York, USA and China, no multicentre studies have considered the impact of hospital strain and resource allocation on the risk of AKI.

### Clinical course and prognosis

*What is the clinical course and prognosis of COVID-19 AKI?*

#### Recommendations

1. *We recommend that patients be monitored for AKI throughout their hospital course and be followed for recovery post-discharge (evidence level: 1B)*.
2. *When feasible, renal histology, particularly in cases of heavy proteinuria, may help to differentiate potential causes of AKI (not graded)*.

#### Rationale

Geographic and regional differences in the course and outcomes of COVID-19 AKI are recognized. However, the influence of resource limitations has not been well described. In general, patients with COVID-19 who develop AKI are more likely to be admitted to the ICU and to require mechanical ventilation and vasopressors than patients who do not develop AKI. Few studies have explored the temporal relationship between the onset or severity of SARS-CoV-2 infection and the development of AKI. Although one study reported that approximately one-third of patients presented either with AKI or developed AKI within 24 h of presentation^16^, another study reported a considerable delay in the manifestation of AKI in patients with COVID-19 (median of 15 days from presentation)^10^, which potentially differentiates COVID-19 AKI from AKI caused by other systemic infections. A temporal association of COVID-19 AKI with intubation has been observed; however, the extent to which these temporal relationships are related to disease progression, organ crosstalk or associated with interventions such as peri-intubation haemodynamic changes is unclear^16,17^.

Available reports indicate that rhabdomyolysis occurs in 7–20% of patients with evidence of COVID-19 AKI^8,17^. Hyperkalaemia has been noted in 23% of patients with COVID-19 AKI, and is often associated with metabolic acidosis^17,69^. As mentioned earlier, a large proportion of patients, particularly those who were critically ill and/or had overt AKI, had evidence of haematuria and proteinuria^8,16,17,64^. Histological evaluation from autopsy series and biopsy case reports of patients with heavy proteinuria have identified several different patterns of injury — collapsing glomerulopathy, proximal tubule injury and microangiopathy with microthrombi — that have the potential to inform subsequent management^23–25,27,32,70^. Of note, various factors specific to COVID-19, including the use of mechanical ventilation, anticoagulation requirements, and logistical complexities given the risk of viral transmission, make renal biopsies difficult to obtain in patients with suspected AKI.

The duration of COVID-19 AKI is poorly understood, and only one study has reported recovery of kidney function^8^. The mortality of COVID-19 AKI has been reported to be between 35% and 80% with rates as high as 75–90% among patients requiring RRT, serving as an independent risk factor for all-cause in-hospital death in patients with COVID-19 (refs^5,8,16,17,60^).

### Research recommendations

1. Future studies should consider the impact of geographical variation, differences in health-care systems, the influence of hospital capacity, the preparedness of health-care systems and social determinants on the epidemiology of COVID-19 AKI, including analysis of how these factors influence risk factors, management of disease and outcomes.
2. Future studies should incorporate information about the proportion of different comorbidities in patients with and without AKI, including potential risk factors for the development of COVID-19 AKI before and after hospital admission.
3. Future studies should determine different phenotypes of COVID-19 AKI based on clinical presentation at diagnosis, patterns of injury, duration and course of AKI, and progression to CKD.
4. The severity of COVID-19 disease at AKI diagnosis and the interventions that have been used for the management of the individual should be reported when describing COVID-19 AKI.
5. The relationship between markers of systemic disease (for example, ferritin, D-dimers, non-respiratory organ failure) and the severity of pulmonary disease to the development, course and outcomes of COVID-19 AKI warrants further study. Risk factors for developing severe AKI (stage 3 AKI or requiring RRT initiation) need to be explored to identify approaches to prevent AKI.
6. The mechanism, timing and clinical implications of traditional markers of AKI (proteinuria and haematuria) as well as novel biomarkers for the diagnosis and prognosis of AKI need to be studied and correlated with markers of systemic disease.
7. Kidney recovery and mortality should be assessed at ICU and hospital discharge. Post-hospitalization outcomes and long-term renal recovery data should be evaluated across different countries.

## Prevention and management of AKI

### Standard-of-care strategies

*What standard-of-care strategies are applicable to the prevention and management of AKI in patients with COVID-19?*

#### Recommendations

1. *Strategies based on KDIGO and other relevant guidelines are appropriate for risk- and stage-based prevention and management of COVID-19 AKI* (Table 2) *(not graded)*.

**Table 2:** Potential management strategies for COVID-19 AKI

Therapy	Rationale	Recommendation
***Standard measures***		
Standard measures based on AKI risk and stage	Prevention and management depend on the risk and stage of AKI	Strategies based on KDIGO and other relevant guidelines are appropriate for risk- and stage-based prevention and management of COVID-19 AKI (ungraded)
Measurement of kidney function	The measurement of kidney function is necessary for precise clinical assessment of risk and stage of AKI. Serum creatinine and urine output are the current gold standards for the evaluation of kidney function, although neither is kidney specific or sensitive for detection of early kidney injury	We recommend monitoring kidney function using a minimum serum creatinine and urine output with careful consideration of the limitations of both (evidence level: 1B)
Haemodynamic optimization	Hypovolaemia, hypotension, and vasoplegia may occur in patients with COVID-19. Fluid and vasopressor resuscitation using dynamic assessment of cardiovascular status may reduce the risk of renal injury and respiratory failure	We recommend individualized fluid and haemodynamic management based on dynamic assessment of cardiovascular status (evidence level: 1B)
Fluid management	The composition of crystalloids for volume expansion is important. Individual trials in non-COVID patients have shown reduced risk of AKI with use of balanced fluids for initial volume expansion, especially in sepsis	We recommend using balanced crystalloids as initial management for expansion of intravascular volume in patients at risk of or with COVID-19 AKI unless an indication for other fluids exists (evidence level: 1A)
Glucose management	Insulin resistance and a hypercatabolic state are common in COVID-19 and contribute to hyperglycaemia	We suggest monitoring for hyperglycaemia and use of intensive glucose-lowering strategies in high-risk patients (evidence level: 2C)
Nephrotoxin management	Nephrotoxins are frequently prescribed in patients with COVID-19. The risks and benefits of these medications and their alternatives need to be closely and frequently assessed. This includes assessment of NSAID use	We recommend limiting nephrotoxic drug exposure where possible and with careful monitoring when nephrotoxins are required (evidence level: 1B)
Use of contrast media	Some studies have challenged the relevance of contrast media toxicity in critically ill patients; furthermore, sodium bicarbonate and N-acetylcysteine have not been shown to prevent contrast-media-associated AKI	We recommend optimization of intravascular volume status as the only specific intervention to prevent contrast-media-associated AKI (evidence level: 1A)
***Experimental strategies***		
Antivirals	Some evidence suggests that direct viral infiltration of tubular cells and podocytes has an impact on tubule function and glomerular filtration	Evidence that antivirals may reduce the risk of COVID-19 AKI is indirect and limited
Immunomodulatory agents (e.g. hydroxychloroquine, corticosteroids, tocilizumab, sarilumab, anakinra, imatinib, dasatinib, ciclosporin, immunoglobulins, baricitinib)	SARS-CoV-2 infection can induce the release of IL-1, IL-6, TNF and other cytokines, as well as secondary HLH. Immunomodulatory agents have the potential to attenuate cytokine production or block cytokine-receptor activation and inhibit autophagy and lysosomal activity to modulate inflammation in host cells	Existing data on immunomodulation in COVID-19 do not show an impact on the development or progression of AKI
Systemic anticoagulation	Thrombi in the renal microcirculation may contribute to the development of AKI	No data are available to show that anticoagulation strategies reduce the risk of AKI or mitigate AKI progression. Systemic anticoagulation may be needed to maintain filter patency during RRT
Statins	Statins inhibit the production of pro-inflammatory cytokines (e.g. TNF, IL-10, IL-6 and IL-8) and the activation and proliferation of T cells, potentially leading to immunomodulation	No data are available to show that statins reduce the risk of AKI or mitigate progression
ACE-I and/or ARBs	ACE-I and ARBs increase ACE2 levels and may rescue cellular ACE2 activity	The impact of RAAS inhibitors on the development or prevention of COVID-19 AKI is uncertain
NSAIDs	Anti-inflammatory properties	Effect unknown
Recombinant ACE2	Potential to neutralize the SARS-CoV-2 and rescue cellular ACE2 activity	Under investigation
Serine inhibitors	Blockage of transmembrane protease serine 2 activity and prevention of viral infiltration	Under investigation

ACE, angiotensin-converting-enzyme inhibitor; ACE2, angiotensin converting enzyme 2; AKI, acute kidney injury; ARB, angiotensin-receptor binder; HLH, haemophagocytic lymphohistiocytosis; JAK, Janus kinase; KDIGO; Kidney Disease: Improving Global Outcomes; RAAS, renin–angiotensin–aldosterone system; RRT, renal replacement therapy.
2. *We recommend individualized fluid and haemodynamic management based on dynamic assessment of cardiovascular status for critically ill patients with COVID-19 (evidence level: 1B)*.
3. *We recommend using balanced crystalloids as initial management for the expansion of intravascular volume in patients at risk of AKI or with AKI unless a specific indication exists for the use of other fluids (evidence level: 1A)*.
4. *We recommend limiting nephrotoxic drug exposure where possible and with careful monitoring when nephrotoxins are required (evidence level: 1B)*.

#### Rationale

**Fig. 2:**
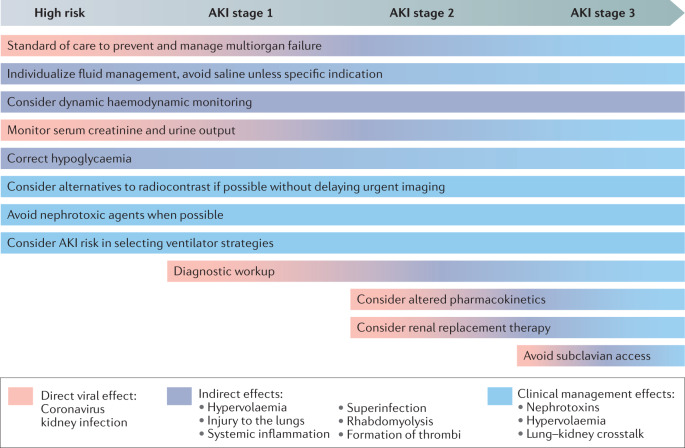
Stage-based management of COVID-19 AKI. The pathogenesis of acute kidney injury (AKI) in patients with COVID-19 (COVID-19 AKI) likely involves direct viral effects, indirect effects and sequelae from disease management. There is no specific evidence to suggest that COVID-19 AKI should be managed differently from other causes of AKI in critically ill patients; however, the possible underlying disease mechanisms should be taken into account when considering approaches to the management of COVID-19 AKI throughout the disease course. Adapted with permission from ref.^61^, Elsevier, and Acute Disease Quality Initiative 25, www.ADQI.org, CC BY 2.0 (https://creativecommons.org/licenses/by/2.0/).

No specific evidence is available to suggest that COVID-19 AKI should be managed differently from other causes of AKI in critically ill patients, and indeed few recommendations for AKI are aetiology specific. Thus, most of the measures recommended by KDIGO and other relevant guidelines are appropriate for patients with COVID-19 (refs^61,71^) (Table 2 and Fig. 2).

Hypovolaemia is common in early COVID-19 and hence individualized fluid management is critical^72^. A randomized clinical trial (RCT) demonstrated that fluid and vasopressor resuscitation based on dynamic haemodynamic assessment may reduce the risk of AKI and respiratory failure in patients with septic shock^73^. We suggest that a similar strategy may be valuable in patients with COVID-19 to reduce the risk of COVID-19 AKI.

Since the publication of the KDIGO guidelines for AKI in 2012 (ref.^61^), more evidence has emerged regarding the importance of the composition of crystalloids used for volume expansion^74–77^. The Isotonic Solutions and Major Adverse Renal Events Trial (SMART) demonstrated that compared with saline, use of balanced crystalloids decreased the composite outcome of death, new RRT, or persistent kidney dysfunction among critically ill adults, with the largest effect observed among patients with sepsis^78^. This beneficial effect was replicated in non-critically ill patients and in the perioperative setting^75,76^. However, three systematic reviews of studies in adults and children did not demonstrate reduced rates of AKI or mortality with balanced crystalloids compared with saline in pooled analyses^79–81^. Given the possible harm caused by the use of 0.9% saline we recommend using balanced fluids, unless there is an indication for other types of fluid (e.g. saline 0.9% for hypochloraemic hypovolaemia).

Several strategies have also emerged for the prevention or mitigation of drug-associated AKI, the most important one being drug stewardship. Single- and multi-centre collaborative studies that have used electronic health records to identify children exposed to ≥3 nephrotoxic drugs led to a sustained decrease in AKI^40,82,83^. The use of multiple nephrotoxic medications is common among patients with COVID-19 who are at a high risk of AKI, so drug stewardship is particularly important.

### COVID-19-specific strategies

*What COVID-19-specific interventions are potentially beneficial for the prevention and management of AKI?*

#### Recommendations

1. *Patients with COVID-19 AKI should be treated using the KDIGO-based standard of care (not graded)*.
2. *We suggest that ventilator strategies in patients with COVID-19 be selected to reduce the risk of AKI when possible (evidence level: 2C)*.

#### Rationale

In the absence of a specific treatment for COVID-19 AKI, management should follow current consensus recommendations for AKI (Table 2). The role of antivirals, immunomodulatory agents (including corticosteroids), renin–angiotensin inhibitors, statins and anticoagulants in the prevention and/or mitigation of AKI remains unknown^2,83–88^ (Table 2). As for patients with other forms of ARDS, lung-protective mechanical ventilation strategies are recommended for patients with severe COVID-19. However, for the reasons noted above, excessive PEEP might result in high systemic venous pressure and a reduction in kidney perfusion and glomerular filtration^54^. Therefore, individualization of PEEP with consideration of its risks and benefits is recommended; the optimal ventilation strategy depends on the degree and extent of airspace disease and compliance of the lungs^89^. Existing data suggest that prone positioning in respiratory failure does not impact the risk of AKI^90^.

Of note, the Adaptive Covid-19 Treatment Trial (ACTT-1) reported that compared with placebo, treatment with remdesivir shortened recovery time among adult patients hospitalized with COVID-19 and evidence of lower respiratory tract infection^84^. In addition, the Recovery trial demonstrated that use of dexamethasone reduced 28-day mortality among hospitalized patients with COVID-19, with the strongest effect seen in patients receiving either invasive mechanical ventilation or oxygen therapy alone at randomization^84,91^. Neither of these trials reported effects on renal function although more details may be available in time.

### AKI-specific treatment considerations

*What interventions in the management of COVID-19 should be modified in patients with AKI?*

#### Recommendations

1. *We recommend that the altered pharmacokinetics and renal effects of COVID-19-specific therapeutics are considered when prescribing and adjusting dosage (evidence level: 1C)*.
2. *COVID-19 is associated with malnutrition; however, whether patients with COVID-19 AKI have specific nutritional requirements is unclear (not graded)*.

#### Rationale

Several drugs or their metabolites that have been proposed for use in patients with COVID-19 are excreted and/or metabolized via the kidneys and require dose adjustment or are contraindicated in patients with impaired kidney function or during RRT. In addition, other conventional therapies, such as antibiotics or anticoagulants, have altered pharmacokinetics in patients with AKI (Supplementary Table 1). Patients with COVID-19 are at risk of malnutrition due to various factors such as prolonged immobilization, catabolic changes and reduced food intake^53,92^; however, no dedicated studies on nutritional management in patients with COVID-19 AKI exist. Therefore, current consensus recommendations for the nutritional management of critically ill patients should be followed^61,93–95^. Protein intake should be gradually increased to 1.3–1.5 g/kg per day in patients with AKI who are not on RRT; 1.0–1.5 g/kg per day for patients on intermittent RRT and up to 1.7 g/kg per day for patients on continuous RRT (CRRT). Early enteral feeding is preferred over parenteral nutrition, and the prone position is not a contraindication to enteral feeding^61,96^.

### Research recommendations

1. Determine the role of antivirals, steroids and systemic anticoagulants in the development and progression of AKI.
2. Determine the pharmacokinetics of antivirals and immunomodulatory drugs during different phases of AKI and progression to acute kidney disease (deteriorating, maintenance and recovery) and with different types of RRT.
3. Explore the nutritional status and energy expenditure of patients with COVID-19 AKI, and determine strategies for their optimal nutritional management according to RRT status.

## Renal replacement therapy

### Patient-specific considerations

*If adequate RRT resources are available, are there patient-specific considerations with respect to vascular access, timing, modality, or dose of acute RRT for patients with COVID-19 AKI?*

#### Recommendations

1. *We recommend that the use of ultrasound for insertion of vascular access and RRT dose delivery remain based on KDIGO AKI guidelines (evidence level: 1A)*.
2. *Timing of RRT initiation, vascular access site and modality of acute RRT should be based on patient needs, local expertise and the availability of staff and equipment* (Tables 3,4) *(not graded)*.

**Table 3:** Recommendations for RRT use in patients with COVID-19 AKI

Considerations	RRT management for COVID-19 AKI	RRT management during a period of increased RRT demand (RRT surge)
RRT indications	Consider acute RRT when metabolic and fluid demands exceed total kidney capacity Consider the broader clinical context and conditions that can be modified by RRT rather than BUN or creatinine alone when determining the need for RRT initiation	Consider a judicious and safe use of intravenous bicarbonate, potassium binding resins and diuretics to forestall RRT initiation RRT should be initiated immediately if there is a failure of conservative measures or clinical deterioration
Modality	Selection of modality should be based on patient needs, local expertise and availability of staff and equipment Prolonged modes of RRT (CRRT, PIRRT, SLED or PD) should be considered for haemodynamically unstable patients, those with marked fluid overload, or in whom shifts in fluid balance are poorly tolerated CVVHD or CVVHDF modality and minimizing post-filter replacement fluid in patients who are on CRRT will decrease the filtration fraction and reduce the risk of circuit clotting	Modality choice may be affected by the supply of disposable materials (dialyzer filters, machine tubing sets, dialysis solutions and anticoagulation medications), machine availability and the availability of appropriately trained staff to operate machines and safely deliver RRT Advantages of PIRRT or IHD may include a reduced need for anticoagulation and shorter duration of therapy session, thereby optimizing machine and human resources to increase the number of patients who can receive RRT per day In the event of limited machine availability, consider shorter durations of IHD or use of CRRT machines for PIRRT (i.e. in a shift-based approach) If IHD or CRRT machine availability is limited, consider use of acute PD, as PD requires relatively less equipment, infrastructure and resources without a need for RRT-related anticoagulation
RRT dose	CRRT: delivered effluent flow rate of 20–25 ml/kg/h (prescribed dose of 25–30 ml/kg/h) IHD or PIRRT: minimum three times per week (alternate days) Interruption of prolonged RRT modality (CRRT, PIRRT or SLED) sessions due to circuit clotting can have a substantial impact on the actual delivered dose and the dose may therefore need to be adjusted to account for this disruption	Consider using lower than usual flow rates once metabolic control has been achieved if concerns exist about the availability of consumables (e.g. filters or dialysate solutions) If shorter durations of IHD or CRRT machines for PIRRT are prescribed or required, we recommend that appropriate adjustments are made in fluid removal targets and RRT dose to achieve appropriate fluid balance targets and metabolic control (e.g. an increase in effluent dose)
Vascular access	Right IJ is the preferred site Prone position, obesity and hypercoagulability may affect vascular access performance	No anticipated differences in preferred vascular access sites during an RRT surge Develop local expertise and teams for acute PD catheter insertion (ICU bedside versus operating room)

AKI, acute kidney injury; BUN, blood urea nitrogen; CRRT, continuous renal replacement therapy; CVVHD, continuous veno-venous haemodialysis; CVVHDF, continuous veno-venous haemodiafiltration; ICU, intensive care unit; IHD, intermittent haemodialysis; IJ, internal jugular; PD, peritoneal dialysis; PIRRT, prolonged intermittent renal replacement therapy; RRT, renal replacement therapy; SLED, slow-low efficiency dialysis.

**Table 4:** RRT modality options for patients with COVID-19 AKI

Modality	Advantages in COVID-19 AKI	Disadvantages in COVID-19 AKI
IHD	Widely available Allows treatment of several patients with the same machine in a given day Higher blood flow may reduce risk of clotting	Less effective in reaching daily fluid balance goals Can lead to or exacerbate haemodynamic instability Usually requires a dedicated HD nurse or other staff in addition to an ICU nurse (increasing staff exposure to the isolation environment)
PIRRT: IHD or CRRT	Less likely than other modalities to exacerbate haemodynamic instability Allows treatment of several patients with the same machine in a given day Option for higher blood flow, which may reduce risk of circuit clotting	Not as widely available as other modalities (i.e. hospital protocols are not widely established) Given the procoagulant nature of COVID-19, systemic anticoagulation may be necessary Challenges and uncertainty of drug dosing, especially for antimicrobial and/or COVID-19 therapeutics
CRRT	Achieves steady-state control of small solutes and acid-base status Least likely to exacerbate haemodynamic instability Easy to achieve net negative fluid balance and achieve fluid balance targets with greater haemodynamic stability Can often be performed by the patient’s bedside in the ICU, limiting staff contact with the isolation environment	Not as widely available as other modalities outside of resource-rich settings or tertiary centres Requires one machine per patient per day Requires ICU settings and may require 1:1 nursing ratio depending on institutional policies Given the procoagulable nature of COVID-19, anticoagulation is recommended and may require systemic therapeutic anticoagulation Increased frequency circuit clotting may lead to a lower delivered dose, inability to achieve fluid balance targets and increased resource utilization (which may have supply chain impacts)
PD	Widely available No circuit clotting concerns No venous access required Less likely to exacerbate haemodynamic instability Less nursing exposure with the use of automated cycler	May be more challenging in patients in prone positions Risk of peri-catheter leaks Protocols and policies for acute PD are not available at all sites. Requires technical expertise to place catheters May require rapid implementation of training regimen for renal nurses and clinicians

AKI, acute kidney injury; CRRT, continuous renal replacement therapy; CVVHD, continuous veno-venous haemodialysis; CVVHDF, continuous veno-venous haemodiafiltration; ICU, intensive care unit; IHD, intermittent haemodialysis; PIRRT, prolonged intermittent renal replacement therapy; PD, peritoneal dialysis; SLED, slow-low efficiency dialysis; RRT, renal replacement therapy.
3. *As COVID-19 often induces a hypercoagulable state, if using CRRT, we suggest use of continuous veno-venous haemodialysis or continuous veno-venous haemodiafiltration to decrease filtration fraction and reduce the risk of circuit clotting (evidence level: 2C)*.

#### Rationale

A number of clinical trials and meta-analyses of RRT initiation strategies in critically ill patients have demonstrated no difference in mortality or renal recovery associated with initiation of RRT in the absence of emergent indications^97–100^. The decision to initiate acute RRT in patients with COVID-19 AKI should therefore be individualized, and clinical context should be considered (e.g. initiation of RRT for volume management in patients with severe hypoxaemia) and not based solely on AKI stage or degree of renal function^61,101^. Selection of RRT modality will depend on local availability and resources, as no clear benefit with any specific RRT modality is known. However, continuous therapies may be better tolerated in patients with haemodynamic instability and facilitate improved volume and nutrition management, which are important in the management of patients with COVID-19 (ref.^61^).

The dose of RRT should be based on KDIGO recommendations and be adjusted in response to changes in clinical, physiological and/or metabolic status^61,102,103^. In patients with COVID-19, coagulopathy resulting in circuit clotting can interrupt prolonged RRT sessions and substantially affect the dose delivered. This complication of COVID-19 may require the RRT prescription to be adjusted^104,105^. If CRRT is used, a reduction in the filtration fraction may reduce circuit clotting^106^. Acute peritoneal dialysis (PD) might also be an effective option for patients who are unable to receive anticoagulants^107–110^.

The choice between jugular or femoral sites for vascular access in patients with COVID-19 is based on the experience and preference of the clinician. For patients with a body mass index >28 kg/m^2^, internal jugular (IJ) sites have lowest infection rates^111,112^. Higher rates of vascular access dysfunction have been observed with the use of the left IJ, compared with the right IJ or femoral sites, but many cases of left IJ access dysfunction probably result from the inadequate depth of the catheter tip at the left IJ site^61,111,113^. The use of ultrasound for the placement of IJ vascular access increases the likelihood of successful catheter placement, with reduced complications and time required for the procedure.

### Anticoagulation strategies

*What is the optimal anticoagulation strategy for acute RRT in COVID-19 AKI?*

#### Recommendations

1. *We recommend that patients with COVID-19 AKI receive anticoagulation agents during extracorporeal RRT (evidence level: 1C)*.
2. *We suggest RRT that circuit performance be closely monitored to ensure maximal circuit patency as the initial anticoagulation strategy may not be effective in all patients; we also recommend that each centre establish a stepwise escalation and/or alternative plans for RRT anticoagulation (evidence level: 2C)*.

#### Rationale

KDIGO recommends the use of anticoagulation for CRRT unless it is contraindicated or the patient is already receiving systemic anticoagulation^61^. COVID-19 induces a hypercoagulable state in many patients, which can result in premature extra-corporeal RRT circuit failure^114–117^. Additionally, an anonymized survey of the ADQI faculty involved in this Consensus Statement revealed that 64% of respondents identified a high rate of circuit loss or clotting during RRT in patients with COVID-19 as an issue requiring “major revision of treatment and anticoagulation protocols” and/or “significantly compromising patient care even after optimization of anticoagulation,” compared with only 3 of 25 (12%) who reported no differences in frequency of RRT circuit loss compared with that observed in patients without COVID-19.

Owing to the hypercoagulable state of patients with COVID-19, therapeutic, titratable, pharmacological anticoagulation has been used more often during RRT to reduce the risk of filter clotting. However, no studies are available to guide the selection of an anticoagulation strategy. Several anticoagulation strategies can be used with a broad array of extra-corporeal RRT therapies in patients with COVID-19, including regional citrate anticoagulation in CRRT, therapeutic, titratable anticoagulation with unfractionated or low-molecular-weight heparins, direct thrombin inhibitors and combinations of these approaches. Change in RRT modality to intermittent haemodialysis (IHD), prolonged intermittent RRT (PIRRT), or acute PD should be considered in patients with persistent circuit clotting during CRRT despite anticoagulation.

Unplanned interruptions due to circuit failures with CRRT, PIRRT or IHD will increase the consumption rate of disposable supplies, increase the risk of exposure of nursing and other staff to infection, increase the risks of inadequate electrolyte, acid-base, fluid balance control, and influence medication pharmacokinetics. Therefore, it is important to strive to maximize the utility of any RRT circuit in order to conserve supplies, as supply chains can become challenged and limited in the context of a pandemic. During an increase in RRT demand, monitoring CRRT circuit lifespan is of paramount importance at both the individual and the organizational level. If poor performance is noted, we suggest prompt implementation of a stepwise approach to anticoagulation to minimize the supply consumption and conserve resources.

### Surge planning

*What are the key considerations when planning for a sudden surge in acute RRT demand?*

#### Recommendations

1. *A coordinated response to an increase in RRT demand and/or supply chain failure at an organizational, regional and national level is needed (not graded)*.
2. *Consider adjustments to RRT modality, indications, anticoagulation and dose as part of a local response to an imbalance in supply and/or demand to conserve scarce resources and deliver effective therapy to the greatest number of patients (not graded)*.

#### Rationale

**Fig. 3:**
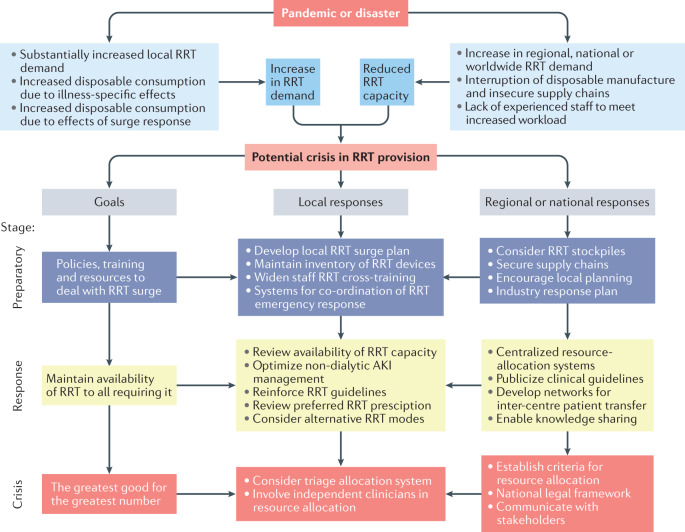
Step-wise plan to prepare for a surge in RRT demand during a pandemic or disaster. A sudden spike in cases of COVID-19 disease might cause unforeseen shortages of renal replacement therapy (RRT) devices and/or RRT disposables and fluids. In addition, supply chain security might be compromised, further contributing to local shortages. As part of a local surge response, use of a wider variety of acute RRT modalities may be needed to maximize the number of patients who can receive RRT. Adapted from Acute Disease Quality Initiative 25, www.ADQI.org, CC BY 2.0 (https://creativecommons.org/licenses/by/2.0/).

In any situation with a rapid increase in ICU demand, it is possible that the provision of RRT may be limited. To prepare for such situations, we recommend that health systems create, maintain and periodically update an RRT ‘surge plan’ (Fig. 3). A sudden spike in cases of COVID-19 or future entities might cause unforeseen shortages of RRT devices and/or RRT disposables and fluids^118^. Additionally, supply chain security may be compromised, further augmenting local shortages^119,120^. As part of a local surge response, use of a wider variety of acute RRT modalities (CRRT, PIRRT, IHD and acute PD) may be needed to maximize the number of patients who can receive RRT. Several institutions reported having to implement acute PD during the initial surge in COVID-19 cases^108,110,121–124^. In the preparatory and early response phase, hospitals and regional health systems should maintain an ongoing inventory of available RRT devices, disposable RRT equipment, and RRT fluids, and make projections of demand to optimize resource utilization. In addition, workforce planning and response to a surge in COVID-19 cases should project the need for trained RRT nursing support and ensure that the relevant human resources are available^125^.

Excessive consumption of RRT disposables due both to increased demand and poor circuit performance has the potential to cause critical shortages, compromising the ability to deliver RRT to all who might benefit. Aggressive medical management of electrolyte and acid-base disturbances or fluid overload might negate the need for RRT or forestall RRT initiation, thereby enabling improved allocation of finite RRT resources^126,127^. Also, depending on the type of shortages anticipated (machine or disposables), strategies such as lowering blood flows to reduce citrate consumption, moderating the RRT intensity to conserve fluids or running accelerated RRT at higher clearance to treat more patients per machine could form part of a local response^128^. Finally, earlier transition to IHD with dialysate generated online or acute PD could be valuable options in centres with appropriate resources and expertise^107,108,110^.

A large surge in COVID-19 cases may make delivery of treatment to all who might benefit impossible, despite appropriate planning. Detailed discussion of the ethical and legal considerations in these circumstances is, however, outside the scope of this consensus statement.

### Research recommendations

1. Develop a registry of patients with severe COVID-19 AKI to research whether variations in clinical practice relating to differences in RRT use and circuit performance affect clinical outcomes.
2. Given the specific issues with circuit loss associated with RRT for COVID-19 AKI, we recommend the planning and initiation of prospective RCTs to examine different anticoagulation strategies for CRRT and PIRRT.
3. We recommend implementation of a programme of operational research, including supply chain management, to examine the local, regional and national responses to the RRT supply–demand imbalance during the COVID-19 pandemic and to develop evidence-based strategies for future emergencies.
4. We recommend that in observational studies, the consequences of a delayed RRT and use of alternative, non-standard modalities in response to the lack of RRT capacity be investigated to determine to what extent the policies developed and implemented during the pandemic were safe and effective.

## Extracorporeal blood purification

### Biological rationale

*What is the potential biological rationale for using (non-renal) EBP in critically ill patients with COVID-19?*

1. *Inflammatory cytokines, DAMPs*, pathogen-associated molecular patterns*(PAMPs), including endotoxins and SARS-CoV-2 particles, potentially contribute to the development of multiple organ failure and mortality in critically ill patients with COVID-19*.
2. *EBP techniques have been shown to remove cytokines, DAMPs and PAMPs, including endotoxins and circulating viral particles*.

#### Rationale

**Fig. 4:**
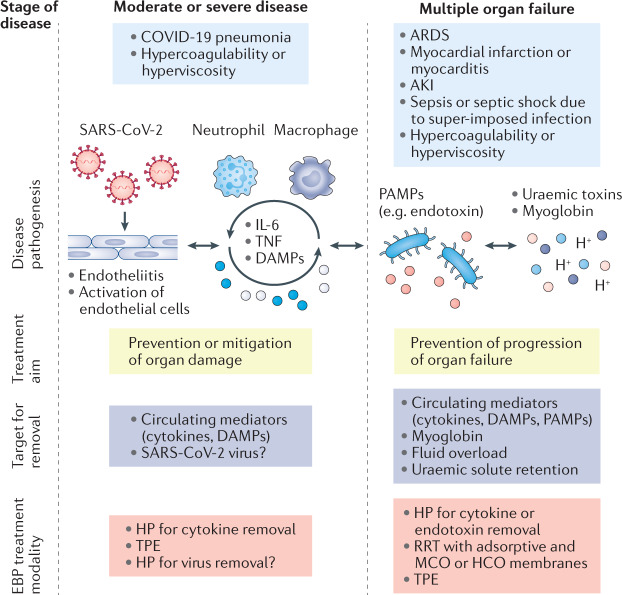
Potential extracorporeal blood purification treatment options based on underlying COVID-19 pathophysiology. Extracorporeal blood purification (EBP) has been proposed as a possible adjuvant therapy for critically ill patients with COVID-19 on the basis that removal of circulating immunomodulatory factors, that might contribute to disease processes and/or the development of multiple organ failure, might improve outcomes. Of note, the efficacy of EBP in patients with COVID-19 and/or COVID-19 AKI has not been tested, and all therapeutic options must therefore be tested in clinical trials in the context of COVID-19. EBP therapies should be considered complementary to pharmacological support. EBP therapies may also be considered in sequence or as separate entities according to current evidence or pathophysiological rationale, as changes in pathophysiology over the disease course might indicate different treatment approaches. AKI, acute kidney injury; ARDS, acute respiratory distress syndrome; COVID-19, coronavirus disease 2019; DAMPs, damage-associated molecular patterns; HCO, high cut-off; HP, haemoperfusion; MCO, medium cut-off; PAMPs, pathogen-associated molecular patterns; RRT, renal replacement therapy; TPE, therapeutic plasma exchange. Adapted from Acute Disease Quality Initiative 25, www.ADQI.org, CC BY 2.0 (https://creativecommons.org/licenses/by/2.0/).

EBP has been proposed as a possible adjuvant therapy for critically ill patients with COVID-19 on the basis that removal of circulating immunomodulatory mediators might prevent organ damage or mitigate organ failure in patients with COVID-19 (refs^129,130^) (Fig. 4). Multiple organ failure in COVID-19 might result from the propagation of an uncontrolled host immune response involving the release of various immune mediators such as cytokines, DAMPs and PAMPs^35,65,131,132^. In sepsis, this type of uncontrolled immune response is characterized by hyperinflammation, cytokine release, endothelial dysfunction and hypercoagulability^66,133–135^. However, as discussed earlier, cytokine activation is not typically as robust in COVID-19 as it is in SARS and MERS^43–45^, or in patients treated with chimeric antigen receptor T cell therapy or with bacterial sepsis^46,47^. Moreover, the benefits and adverse effects of EBP in patients with COVID-19 have not been formally studied. Thus, we suggest that patients for whom EBP is being considered are selected carefully.

### EBP techniques

*Which EBP techniques can potentially be used to remove circulating molecules implicated in the pathophysiology of COVID-19?*

1. *Haemoperfusion techniques can remove inflammatory molecules, DAMPs and PAMPs, including SARS-CoV-2 particles*.
2. *Therapeutic plasma exchange (TPE) can remove inflammatory mediators and proteins associated with hypercoagulability*.
3. *CRRT with surface-modified AN69 or polymethylmethacrylate membranes can remove target molecules by adsorption, whereas CRRT with medium cut-off or high cut-off membranes can remove target molecules by diffusion or convection*.

#### Rationale

Many health-care agencies have authorized emergency use of various EBP techniques to remove molecules that are potentially causative of the immuno-inflammatory response in critically ill patients with COVID-19. However, these EBP techniques have not yet been formally studied in this patient population (Supplementary Table 2). Haemoperfusion sorbents might target the removal of virus particles, cytokines and DAMPs in patients with high endotoxin levels^136–140^. In a small RCT of patients with septic shock (EUPHAS), the use of haemoperfusion was associated with improved organ function and a survival benefit^141^; however, a larger RCT (EUPHRATES) failed to confirm these findings^136^. A post hoc analysis of the EUPHRATES trial demonstrated possible therapeutic survival effect in a subgroup of patients with endotoxin activity in a specific range^140^. TPE has been shown in RCTs to improve haemodynamics, induce favourable changes in cytokine profile and improve survival in patients with septic shock^142,143^. Removal of inflammatory cytokines with TPE could, in theory, confer some benefit in patients with COVID-19 with hyperinflammation and hypercoagulability^144^. CRRT with medium cut-off, high cut-off or adsorptive membranes can remove cytokines or myoglobin and potentially prevent myoglobin-induced AKI^145,146^.

### Criteria for EBP use

*What are possible biological and/or clinical criteria for initiating, monitoring, and discontinuing EBP in critically ill patients with COVID-19?*

#### Recommendations

1. *No consensus exists on the use or thresholds of specific biological and clinical criteria for initiating, monitoring or discontinuing EBP in critically ill patients with COVID-19 (not graded)*.

#### Rationale

If used, EBP therapies should be selected on the basis of the pathophysiology they are designed to target. Numerous clinical criteria, including body temperature, haemodynamic status, need for vasopressor support, respiratory status and oxygenation, multiorgan failure score, cardiac and kidney function, as well as laboratory parameters such as lymphocyte counts, concentration of cytokines, ferritin, lactate dehydrogenase, D-dimers, monocytic expression of HLA, myoglobin, troponin, C-reactive protein, endotoxin activity, procalcitonin and culture results may be useful in evaluating the suitability of a patient for initiation of EBP. However, the precise indication for EBP in patients with COVID-19 remains to be determined. EBP for endotoxin removal has been generally applied for 48 consecutive hours and for 72 h for cytokine removal in studies of septic patients and in ongoing COVID-19 trials^140,146–149^. However, there are limited data regarding the timing of initiation or duration of use of these therapies, and further studies are needed.

### Research recommendations

1. Future trials should measure the ability of EBP to remove target molecules, including assessment of their kinetics, to confirm the pathophysiological rationale for use of EBP in critically ill patients with COVID-19.
2. Future trials should assess whether use of EBP is associated with improved short-term outcomes, including prevention and mitigation of organ failure.
3. Future trials should assess whether combined or sequential EBP techniques can reach meaningful biological and/or clinical end points.
4. The ability of haemoperfusion to prevent or mitigate organ failure by removal of the SARS-CoV-2 virus in patients with detected viraemia should be investigated.
5. Future studies should validate the biological and clinical parameters that identify individuals who are likely to benefit and respond to EBP, as well as parameters for monitoring and discontinuing treatments.
6. Future studies should evaluate TPE as an alternative for reducing hypercoagulability, hyperviscosity, and hyperinflammation in patients with COVID-19, and also assess the negative consequences of removing potentially beneficial molecules (e.g. removal of protective SARS-CoV-2 antibodies).
7. Future studies should assess the removal of drugs and nutrients during EBP and any resulting potentially negative consequences on patient outcomes.

## Conclusions

Kidney involvement following SARS-CoV-2 infection is more common than initially thought and is associated with morbidity and mortality. The pathophysiology of COVID-19 AKI is probably multifactorial — in line with the pathophysiology of other forms of AKI. Rates of COVID-19 AKI vary considerably between studies and regions, although available evidence suggests an incidence of over 20% in hospitalized patients. Many features, such as risk factors, likely mechanisms and outcomes, are shared between COVID-19 AKI and AKI arising from non-viral causes encountered in the ICU. Thus, many of the treatment recommendations, as well as preventative measures described in this Consensus Statement, are common to both. Considerations for RRT are also similar with the caveat that more aggressive anticoagulant regimes may be needed and that treatment may need to be adjusted to conserve resources in the context of a surge in COVID-19 cases. Given the potential contribution of systemic inflammation to multiorgan failure in COVID-19, the use of extracorporeal therapies may also be considered. Of note, new data on COVID-19 AKI are continually being published and these recommendations may therefore require modifications as new results become available.

## Supplementary information

Supplementary Information

## Glossary

D-dimer: A protein fragment resulting from the breakdown of blood clots. D-dimer levels are used to identify intravascular thrombosis.
Cytokine storm: Uncontrolled cytokine release into the systemic circulation, often associated with defects in immune regulation, either genetic or acquired such as in macrophage activation syndrome.
Positive end-expiratory pressure: (PEEP). Pressure applied to a ventilatory circuit to prevent airway collapse and to increase mean airway pressure, a determinant of oxygenation.
Damage-associated molecular patterns: (DAMPs). Endogenous molecules that can precipitate inflammation often by signalling through specific receptors such as Toll-like receptors. DAMPs frequently arise from injured or dying cells (e.g. myoglobin, HMGB1, uric acid).
Pathogen-associated molecular patterns: (PAMPs). Molecules released from pathogens (especially bacteria) that can precipitate inflammation often signalling through specific receptors such as Toll-like receptors. Common PAMPs include components of the bacterial cell wall (e.g. endotoxin, lipoteichoic acid).
