# Publisher Correction: COVID-19-associated acute kidney injury: consensus report of the 25^th^ Acute Disease Quality Initiative (ADQI) Workgroup

**DOI:** 10.1038/s41581-020-00372-5

**Published:** 2020-11-02

**Authors:** Mitra K. Nadim, Lui G. Forni, Ravindra L. Mehta, Michael J. Connor, Kathleen D. Liu, Marlies Ostermann, Thomas Rimmelé, Alexander Zarbock, Samira Bell, Azra Bihorac, Vincenzo Cantaluppi, Eric Hoste, Faeq Husain-Syed, Michael J. Germain, Stuart L. Goldstein, Shruti Gupta, Michael Joannidis, Kianoush Kashani, Jay L. Koyner, Matthieu Legrand, Nuttha Lumlertgul, Sumit Mohan, Neesh Pannu, Zhiyong Peng, Xose L. Perez-Fernandez, Peter Pickkers, John Prowle, Thiago Reis, Nattachai Srisawat, Ashita Tolwani, Anitha Vijayan, Gianluca Villa, Li Yang, Claudio Ronco, John A. Kellum

**Affiliations:** 1 Division of Nephrology and Hypertension, Department of Medicine, Keck School of Medicine, University of Southern California, Los Angeles, CA USA; 2 Department of Clinical and Experimental Medicine, Faculty of Health Sciences, University of Surrey, Guildford, UK; 3 Intensive Care Unit, Royal Surrey County Hospital NHS Foundation Trust, Guildford, UK; 4 Division of Nephrology, Department of Medicine, University of California, San Diego, CA USA; 5 Divisions of Pulmonary, Allergy, Critical Care, & Sleep Medicine, Division of Renal Medicine, Department of Medicine, Emory University School of Medicine, Atlanta, GA USA; 6 Divisions of Nephrology and Critical Care Medicine, Departments of Medicine and Anesthesia, University of California, San Francisco, CA USA; 7 Department of Intensive Care, Guy’s & St Thomas’ NHS Foundation Hospital, London, UK; 8 Department of Anesthesiology and Intensive Care Medicine, Edouard Herriot Hospital, Hospices Civils de Lyon, Lyon, France; 9 Department of Anesthesiology, Intensive Care and Pain Medicine, University Hospital Münster, Münster, Germany; 10 Division of Population Health and Genomics, School of Medicine, University of Dundee, Dundee, UK; 11 Department of Medicine, University of Florida, Gainesville, FL USA; 12 Nephrology and Kidney Transplantation Unit, Department of Translational Medicine, University of Piemonte Orientale, Novara, Italy; 13 Intensive Care Unit, Ghent University Hospital, Ghent University, Ghent, Belgium; 14 Division of Nephrology, Pulmonology and Critical Care Medicine, Department of Medicine II, University Hospital Giessen and Marburg, Giessen, Germany; 15 Division of Nephrology, Renal Transplant Associates of New England, Baystate Medical Center U Mass Medical School, Springfield, MA USA; 16 Division of Nephrology and Hypertension, Cincinnati Children’s Hospital Medical Center, Cincinnati, OH USA; 17 Division of Renal Medicine, Brigham and Women’s Hospital, Boston, MA USA; 18 Division of Intensive Care and Emergency Medicine, Department of Medicine, Medical University of Innsbruck, Innsbruck, Austria; 19 Division of Nephrology and Hypertension, Division of Pulmonary and Critical Care Medicine, Department of Medicine, Mayo Clinic, Rochester, MN USA; 20 Division of Nephrology, Department of Medicine, University of Chicago, Chicago, IL USA; 21 Department of Anesthesiology and Perioperative Care, University of California San Francisco, San Francisco, CA USA; 22 Division of Nephrology, Excellence Center for Critical Care Nephrology, Critical Care Nephrology Research Unit, King Chulalongkorn Memorial Hospital, Chulalongkorn University, Bangkok, Thailand; 23 Department of Medicine, Division of Nephrology, Columbia University College of Physicians & Surgeons and New York Presbyterian Hospital, New York, NY USA; 24 Department of Epidemiology, Mailman School of Public Health, Columbia University, New York, NY USA; 25 Division of Critical Care Medicine, Faculty of Medicine and Dentistry, University of Alberta, Edmonton, Alberta Canada; 26 Division of Critical Care Medicine, Zhongnan Hospital of Wuhan University, Wuhan, China; 27 Servei de Medicina Intensiva, Hospital Universitari de Bellvitge, L’Hospitalet de Llobregat, Barcelona, Spain; 28 Department of Intensive Care Medicine, Radboudumc, Nijmegen, The Netherlands; 29 Critical Care and Peri-operative Medicine Research Group, William Harvey Research Institute, Queen Mary University of London, London, UK; 30 Department of Nephrology, Dialysis and Transplantation, San Bortolo Hospital, International Renal Research Institute of Vicenza, Vicenza, Italy; 31 Department of Nephrology, Clínica de Doenças Renais de Brasília, Brasília, Brazil; 32 Academy of Science, Royal Society of Thailand, Bangkok, Thailand; 33 Division of Nephrology, Department of Medicine, University of Alabama, Birmingham, AL USA; 34 Division of Nephrology, Washington University School of Medicine, St. Louis, MO USA; 35 Section of Anaesthesiology and Intensive Care, Department of Health Science, University of Florence, Florence, Italy; 36 Renal Division, Peking University First Hospital, Beijing, China; 37 Department of Medicine, University of Padova, Padova, Italy; 38 Department of Critical Care Medicine, Center for Critical Care Nephrology, University of Pittsburgh, Pittsburgh, PA USA

*Nature Reviews Nephrology* (2020) 10.1038/s41581-020-00356-5 Published online 15 October 2020

In the version of this article that was originally published online, the text in the red boxes (crisis stage) of Figure 3 was a duplication of the text in the yellow boxes (response stage). This error has been corrected in the HTML and PDF versions of the manuscript.

