# Supplementary material for: COVID-19-associated acute kidney injury: consensus report of the 25th Acute Disease Quality Initiative (ADQI) Workgroup
Source: Nat Rev Nephrol. 2020 Oct 15;16(12):747–64. doi: 10.1038/s41581-020-00356-5 (PMC7561246; doi:10.1038/s41581-020-00356-5)
Supplement: Supplementary file 1 — Supplementary Information [file 41581_2020_356_MOESM1_ESM.pdf]

natureresearch

https://doi.org/10.1038/s41581-020-00356-5

Supplementary information

COVID-19-associated acute kidney injury: consensus report of the 25th Acute Disease Quality Initiative (ADQI) Workgroup

In the format provided by the authors and unedited

Supplementary Box 1 | 25th ADQI workgroup co-chairs and members

Group 1: Epidemiology and diagnosis
- Co-chairs: Alexander Zarbock (Germany), Ravindra L. Mehta (USA)
- Members: Michael J. Germain (USA), Shruti Gupta (USA), Eric Hoste (Belgium), Sumit Mohan (USA), Li Yang (China)

Group 2: Pathophysiology and effects of treatment
- Co-chairs: Kathleen D. Liu (USA), Lui G. Forni (UK)
- Members: Samira Bell (UK), Vincenzo Cantaluppi (Italy), Michael Joannidis (Austria), Jay L. Koyner (USA), Matthieu Legrand (USA)

Group 3: Prevention and management of AKI
- Co-chairs: Marlies Ostermann (UK), John A. Kellum (USA)
- Members: Azra Bihorac (USA), Stuart L. Goldstein (USA), Kianoush Kashani (USA), Zhiyong Peng (China), Nattachai Srisawat (Thailand)

Group 4: Renal replacement therapy
- Co-chairs: Michael J. Connor, Jr. (USA), Mitra K. Nadim (USA)
- Members: Neesh Pannu (Canada), Xose L. Perez-Fernandez (Spain), Peter Pickkers (Netherlands), John Prowle (UK), Anitha Vijayan (USA)

Group 5: Extracorporeal blood purification
- Co-chairs: Thomas Rimmele (France), Claudio Ronco (Italy)
- Members: Faeq Husain-Syed (Germany), Nuttha Lumlertgul (Thailand), Thiago Reis (Brazil), Ashita Tolwani (USA), Gianluca Villa (Italy)

Supplementary Table 1 | Pharmacokinetics, recommended dosage, and potential effects on the kidney of currently proposed COVID-19 drugs.

Drugs	Action	MW	Protein binding	Metabolism and drug–drug interaction	Excretion	Route	Dosing in normal GFR	Dosing in reduced GFR	Dosing in RRT	Potential effects on kidney
**Antiviral drugs**										
Remdesivir	Adenosine analogue, which inhibits RdRp	602.2 Da	88%	Intracellular CYP3A4 inhibitor	• Renal excretion of excipient sulfobuthylether-β-cyclodextrin sodium salt (SBECD)<br>• 74% renal excretion of metabolite GS-441524	IV	200mg (day 1) then 100mg qd (5-10 days)	Not recommended if GFR < 30 ml/min/1.73m^2	NR	• Potential accumulation of the excipient SBECD<br>• Evidence of kidney damage in animal studies (FDA)
Favipiravir	Purine analogue, which inhibits RdRp	157.1 Da	54%	Liver	Mainly renal excretion of inactive metabolite T-705M1s	Oral	1,600mg bd (day 1), then 600mg qd (day 2-5)	Not recommended if GFR < 20 ml/min/1.73m^2	NR	Not reported
Umifenovir (arbidol)	Inhibitor of spike protein/ACE2 interaction	477.4 Da	N/A	Liver and intestine by CYP3A4	Faeces	Oral	200mg tid up to 21 days	No dose adjustment	N/A	Not reported
Lopinavir / ritonavir	Protease inhibitors	1349.7 Da	98-99%	Liver<br>Ritonavir: CYP3A4 inhibitor and substrate	Mainly faeces<br>2% urine	Oral	400/100mg bd	No dose adjustment	No dose adjustment	Case reports of AKI
Camostat mesylate	Serine protease inhibitor	494.5 Da	25.8-28.2%	Hydrolysis by carboxyesterase	89.8-95.6% urine	Oral	200mg tid	N/A	N/A	Not reported
**Anti-malaria drug**										
Hydroxychloroquine / Chloroquine	Viral entry and endocytosis inhibitor	335.9 Da	50%	Liver by CYP3A4	50% urine (16-21% as unchanged drug)	Oral	HCQ: 400-600mg bd (day 1) then 200mg bd (for up to day 5)	No dose adjustment, prescribe with caution	Chloroquine: HD = 50% dose reduction; CRRT = full dose; Hydroxychloquine:	Renal lipidosis mimicking Fabry disease presenting with proteinuria

									HD, CRRT = N/A, use with caution	
**Anti-parasitic drug**										
Ivermectin	Viral protein cargos inhibitor	1735.2 Da	93%	liver	feces	Oral	12mg single dose	No dose adjustment	N/A	Not reported
Anti-inflammatory / immunomodulatory drugs										
Colchicine	Lipid soluble alkaloid	399.4 Da	39%	Liver by CYP3A4 and P-gp1	Mainly hepatobiliary 10-20% urine	Oral	0.5mg bd	Reduce dose by 50% in GFR < 50 ml/min/1.73m^2	0.3mg qd	Rhabdomyolysis
Tocilizumab	Recombinant IL-6 monoclonal antibody	148 kDa	N/A	Proteolysis	Not via renal or hepatic pathways	IV	8mg/kg (up to 800mg) 1-2 doses	No dose adjustment	N/A	Not reported
Sarilumab	Recombinant IL-6 monoclonal antibody	150 kDa	N/A	Proteolysis	Not via renal or hepatic pathways	SC	200mg single dose	No dose adjustment	N/A	Not reported
Anakinra	IL-1 receptor antagonist	17.3 kDa	N/A	N/A	80% urine	IV or SC	5mg/kg (IV) or 200 mg bd (sc)	Reduce dose by 50% or alternate day	N/A	Not reported
Emapalumab	IFN-γ blocking antibody	154.4 kDa	N/A	Proteolysis	Not via renal or hepatic pathways	IV	Day 1: 6mg/kg. Days 4, 7, 10 and 13: 3mg/kg	No dose adjustment	No dose adjustment	Not reported
Interferon	Recombinant interferon	20 kDa	N/A	N/A	N/A	SC	(IFN-beta 1b) 8mU alternate days (14 days)	No dose adjustment, prescribe with caution	Use with caution	Renal TMA
Canakinumab	Human anti-IL-1β monoclonal antibody	145.2 kDa	N/A	N/A	N/A	IV	450 - 700mg single dose	N/A	N/A	Not reported

Bevacizumab	Anti-VEGF recombinant humanized monoclonal antibody	149 kDa	N/A	Proteolysis	Not via renal or hepatic pathways	IV	7.5mg/kg	No dose adjustment	No dose adjustment	Proteinuria; renal TMA
Ruxolitinib	Selective JAK1 and JAK2 inhibitor	306.4 Da	97%	Liver by CYP3A4 substrate	74% urine<br>22% faeces	Oral	5mg bd (14-28 days)	Reduce dose by 50% or avoid if GFR < 59 mL/min/1.73m^2	15-20mg once post HD	Not reported
Baricitinib	Selective JAK1 and JAK2 inhibitor	371.4 Da	50%	Liver<br>CYP3A4 substrate	69% urine<br>15% faeces	Oral	2mg qds (10 days)	If GFR 30-60 ml/min/1.73m^2: reduce to 1mg qd;<br>not recommended if GFR < 30 ml/min/1.73m^2	N/A	Reduce proteinuria in diabetic kidney disease

AKI, acute kidney injury; GFR, glomerular filtration rate; HCQ, hydroxychloroquine; HD, haemodialysis; IFNγ; interferon gamma; IL, interleukin; IV, intravenous; JAK; janus kinase; kDa; kilodalton; MW, molecular weight; N/A, not available; NR: not recommended; P-gp1, P-glycoprotein1; RdRp, RNA-dependent RNA polymerase enzyme; RNA, ribonucleic acid; RRT; renal replacement therapy; SC, subcutaneous; TMA; thrombotic microangiopathy; VEGF, vascular endothelial growth factor.

Supplementary Table 2 | Composition, use and prescription of extracorporeal blood purification using cartridges and RRT membranes.

**Technique and associated considerations**	**Hyperinflammation**	**Gram-negative sepsis; endotoxaemia**	**AKI**	**AKI; increased myoglobin levels; hyperinflammation**	**AKI; hyperinflammation; Gram-negative sepsis; endotoxaemia**
Technique	Haemoperfusion	Haemoperfusion	Haemodialysis, haemofiltration or haemodiafiltration	Haemodialysis (CVVHD for MCO or HCO), haemofiltration or haemodiafiltration	Haemodialysis, haemofiltration or haemodiafiltration
Membrane composition	Porous polymer beads; sorbents	PMX covalently bound to polypropylene–polystyrene fibre	AN69; PMMA; polysulfone	AN69-ST; MCO; HCO	AN69-PEI concentrated; PMMA
Target	Cytokines	Endotoxin	Uraemic toxins; excess fluid; electrolyte and acid–base disorders	Uraemic toxins; excess fluid; electrolyte and acid–base disorders; myoglobin; cytokines	Uraemic toxins; excess fluid; electrolyte and acid–base disorders; cytokines; endotoxin
Mechanism of removal	Adsorption	Adsorption	Filtration (convection and diffusion)	Adsorption (cytokines); filtration (convection and diffusion)	Adsorption (cytokines and endotoxin); filtration (convection and diffusion)
Duration	2–24h	2h	Up to 72h (with the same disposable)	Up to 72h (with the same disposable)	Up to 24h (with the same disposable)
Frequency	1–3 days	2 days	Variable	Variable	1–3 days
Anticoagulation	Citrate or heparin	Citrate or heparin	Citrate or heparin	Citrate or heparin	Citrate or heparin
Blood flow	100–500 ml/min	80–120 ml/min	150–250 ml/min	150–250 ml/min; 80–200 ml/min for MCO or HCO	150–250 ml/min

AKI, acute kidney injury; AN, acrylonitrile; COVID-19, coronavirus disease 2019; CRRT, continuous renal replacement therapy; CVVHD, continuous veno-venous haemodialysis; HCO, high cut-off; HP, hemoperfusion; MCO, medium cut-off; PEI, polyethyleneimine; PMMA, polymethylmethacrylate; PMX, polymyxin B; RRT, renal replacement therapy; SARS-CoV-2, severe acute respiratory syndrome coronavirus 2; ST, surface-treated. Adapted with permission from the Acute Disease Quality Initiative 25, www.ADQI.org.
